# Constructing representative group networks from tractography: lessons from a dynamical approach

**DOI:** 10.3389/fnetp.2024.1457486

**Published:** 2024-11-08

**Authors:** Eleanna Kritikaki, Matteo Mancini, Diana Kyriazis, Natasha Sigala, Simon F. Farmer, Luc Berthouze

**Affiliations:** ^1^ Department of Informatics, University of Sussex, Brighton, United Kingdom; ^2^ Department of Cardiovascular, Endocrine-Metabolic Diseases and Aging, Italian National Institute of Health, Rome, Italy; ^3^ Cardiff University Brain Research Imaging Centre (CUBRIC), Cardiff University, Cardiff, United Kingdom; ^4^ Department of Clinical Neuroscience, Brighton and Sussex Medical School, University of Sussex, Brighton, United Kingdom; ^5^ Department of Neurology, National Hospital for Neurology and Neurosurgery, London, United Kingdom; ^6^ Department of Clinical and Human Neuroscience, UCL Institute of Neurology, London, United Kingdom

**Keywords:** group-representative networks, metastability, synchronisation, tractography, connectome analysis, dynamics

## Abstract

Human group connectome analysis relies on combining individual connectome data to construct a single representative network which can be used to describe brain organisation and identify differences between subject groups. Existing methods adopt different strategies to select the network structural features to be retained or optimised at group level. In the absence of ground truth, however, it is unclear which structural features are the most suitable and how to evaluate the consequences on the group network of applying any given strategy. In this investigation, we consider the impact of defining a connectome as representative if it can recapitulate not just the structure of the individual networks in the cohort tested but also their dynamical behaviour, which we measured using a model of coupled oscillators. We applied the widely used approach of consensus thresholding to a dataset of individual structural connectomes from a healthy adult cohort to construct group networks for a range of thresholds and then identified the most dynamically representative group connectome as that having the least deviation from the individual connectomes given a dynamical measure of the system. We found that our dynamically representative network recaptured aspects of structure for which it did not specifically optimise, with no significant difference to other group connectomes constructed via methods which did optimise for those metrics. Additionally, these other group connectomes were either as dynamically representative as our chosen network or less so. While we suggest that dynamics should be at least one of the criteria for representativeness, given that the brain has evolved under the pressure of carrying out specific functions, our results suggest that the question persists as to which of these criteria are valid and testable.

## 1 Introduction

Diffusion weighted imaging (DWI) lies at the heart of various pipelines for the systematic mapping of the structural connectivity between brain regions (Jones, 2008; Soares et al., 2013). Such approaches have been extensively used to build models of the brain as a network (the human “connectome”). Studies of the human connectome utilise tools from graph theory to describe principles of brain structural organisation (Hagmann et al., 2012). These approaches help elucidate the relationship between brain structure and function in healthy and diseased brain states, facilitating comparisons that can shed important light on the pathophysiology of human brain disorders (Deco and Kringelbach, 2014; Meunier et al., 2010; van den Heuvel and Sporns, 2011).

DWI pipelines aiming to reconstruct the human connectome lead to the creation of *individualised* networks from each of the subjects’ fibre-tracking data. However, a common practice in network neuroscience has been the construction of a representative *group* network by collating structural brain network data from a cohort of individual subjects (Hagmann et al., 2008; van den Heuvel and Sporns, 2011; Cabral et al., 2011; Cabral et al., 2014; Schirmer et al., 2019). This step aims to minimise individual variability and outliers by constructing a “blueprint” brain network that, while still inevitably contaminated by the biases introduced from the processing pipeline (Schilling et al., 2019), is expected to facilitate the accuracy of quantitative analyses. The conclusions to be drawn are thought to be more applicable to the general characteristics of the brain. The aims of the various studies that utilise group brain networks range from characterising the healthy human connectome and describing universal brain topology (Hagmann et al., 2008; van den Heuvel and Sporns, 2011; Betzel and Bassett, 2018) to making meaningful comparisons between pre-defined groups, such as healthy and diseased populations (Wang et al., 2021), and between healthy subjects across a large age range (Buchanan et al., 2020).

In early brain network studies (Hagmann et al., 2008; Cabral et al., 2011; Cabral et al., 2014), averaging each element in the adjacency matrix across all subjects to obtain a group network was a common technique. However, a simple average can be problematic; for example, elements in a few subjects’ matrices that may represent outliers due to noise will inevitably be represented in the average, while the resulting group network will have a much higher binary density than any of the subjects due to inherent cohort variability (e.g., one can imagine “overlapping” different adjacency matrices where different elements are equal to 0 in each matrix).

As such, more sophisticated techniques have been developed to create group brain networks. Most of these can be said to be variants of one of the following three approaches.

In *consensus thresholding* (van den Heuvel and Sporns, 2011; van den Heuvel et al., 2012; Schirmer et al., 2019; Parkes et al., 2023), only those links present in any given percentage of the individuals (the percentage consensus threshold) are retained in the group connectome. However, there is no “consensus” regarding which is the appropriate percentage threshold, leaving this decision to the discretion of the researcher. The choice of a threshold can differentially affect the outcomes of investigations, making comparisons between different studies difficult. For example, in a study by Buchanan et al. (2020), the stringency of the threshold applied had significant consequences on the association of brain network properties with age. de Reus and van den Heuvel (2013) devised a model that helps make an informed choice about a particular threshold based on determining an acceptable trade-off between the assumed false positive and false negative connections in the group network. They suggested that a wide range of thresholds between 30% and 90% would be appropriate. However, their model was based on two assumptions: 1) that most of the variation in the connectomes represents noise; 2) that the level of certainty regarding the existence of a link depends on its prevalence in the cohort (de Reus and van den Heuvel, 2013). Neither of these assumptions is necessarily true because we do not know how much variability exists in the human connectome. Furthermore, consistency in the presence of a connection can easily result from a systematic error. de Reus and van den Heuvel (2013) reported a large variability of global network properties within the “acceptable” threshold range, including characteristic path length, clustering coefficient, and the degree assortativity. Differences in network structure can influence network dynamics, so this variability is expected to significantly affect the dynamical behaviour of networks within a range (Arenas et al., 2008; Rodrigues et al., 2016).

A significant source of bias introduced by consensus thresholding is that in each individual brain network, the probability that a connection exists decays monotonically with anatomical distance (Alexander-Bloch et al., 2013; Betzel and Basset, 2018). Tractography algorithms contribute to this issue due to the premature termination of streamlines (Yeh et al., 2021). The uniform application of a single threshold across all connections (as is the case with consensus thresholding) will result in the reconstructed group network overestimating the presence of short-range connections whilst underestimating the proportion of long-range connections. To mitigate this problem, the *distance-dependent consensus threshold* Betzel et al. (2019) includes the application of a “restricted” rather than a uniform threshold on each link. This “restricted” threshold is calculated based on the length of that connection (see Section 2.7.2. for more detail). This approach results in a group network that closely recapitulates the inter-areal Euclidean distance distributions of the individual networks. Applying this form of threshold has also been shown to better recapitulate several local and global network features compared to uniform consensus thresholding (Betzel et al., 2019), including the network degree, clustering coefficient, and betweenness centrality. However, distance-dependent consensus thresholding operates on the assumption that the long-range connections preserved do indeed represent existent connections and are not spurious links. Furthermore, it cannot be performed on fully weighted brain networks with very high levels of density and hence no meaningful consensus between links, since all, or nearly all, links, are present in all subjects (Betzel et al., 2019). We note here that even if a matrix of inter-areal Euclidean distances is not explicitly used in any given model of the brain (such as the non-spatially embedded model we use here), it is impossible not to be biased by the implicit topological information that is carried in the inter-areal Euclidean distances (which are here used as a proxy for connection lengths). For example, the 50–100 mm bracket of connection lengths between brain regions contains major association fibres which connect different brain lobes: any thresholding strategy with an effect on this length bracket would, therefore, also affect binary interlobular connectivity (for a detailed map of the topology of brain connections of different lengths, see Bajada et al. (2019)). This argument can also be applied to the weights of the links, even if the final model of the brain only deals with binary connectivity. As such, if one decides to threshold a brain network with a strategy based on weights before binarizing, the final binarized network will suffer from a specific bias, which originates from the “weighted topology” of the network. For example, the links with the highest weights in our dataset are links that connect hubs together, corresponding to rich-club connections–data not shown.

The last method of group network construction we describe does rely on weighted connectivity. *Consistency-based thresholding* (Roberts et al., 2016) retains those links with weights that have the lowest variation across the subjects, up to a desired group density. As such, it can deal with even nearly fully connected networks, such as those that can be produced by probabilistic tractography (Roberts et al., 2016) where a consensus cannot be established (i.e., nearly all subjects possess all connections). Networks constructed with this method have been found to better capture the edge length distribution of the original networks, retaining a greater proportion of long-range connections and, consequently, inter-hemispheric connections when compared with a group connectome constructed via thresholding by weight (Roberts et al., 2016). However, the choice of the threshold (density) is still an arbitrary parameter: Mccolgan et al. (2018) showed that different thresholds can impact the detection of important group differences between healthy and diseased groups.

Each of the methods described above utilise different criteria to determine which connections are retained in the group connectome, based on assumptions about which links represent noise and which represent individual variability. Due to the absence of a ground truth connectome, there is no in-principle way to determine the accuracy of these assumptions. As networks are integrated systems, there is also a real possibility that optimising certain aspects of the group connectome may lead to unexpected changes in other aspects, some of which may lead the group connectome to deviate from the cohort in terms of representativeness.

In this investigation, we develop a novel method to choose the most representative group network based on the metastability of the individual networks. Using this method, we test the notion of cohort representativeness by asserting that for a group network to be considered representative of the individuals from which it is constructed, then given a dynamical process running on the network, the resulting behaviour should also be representative of that of the individuals. In what follows, we describe the rationale behind the choice of signature for the dynamical behaviour (Section 2.2), the construction of a set of group networks that served as candidates from which to choose the one that was most dynamically representative (2.3), and the method we used to find the optimum (2.4). Upon choosing the most dynamically representative connectome, we assessed the quality of the fit of its dynamical signature to those of the individual subjects (3.1), and we compared this fit with that of group connectomes constructed with alternative methods (3.2.1). Finally, we also compared the structural representativeness of all the group networks in this study (3.2.2), and we discuss the implications of these findings in Section 4.

## 2 Methods

### 2.1 Simulating network dynamics with the Kuramoto model

To characterise the dynamics of the group and individual (subject) networks, we used a modification of the classical Kuramoto model of synchronisation (Arenas et al., 2008). In the context of the Kuramoto model, each node of the network *i*, representing a brain region, is regarded as a phase oscillator with its own intrinsic frequency— $\omega_{i}$ . Each phase oscillator interacts with the other oscillators in a manner dependent on its phase difference with them, as well as the existence of a structural connection between them in the network.

The equation that describes the time evolution of the phase $\theta_{i}$ of each oscillator in the Kuramoto model is the following (1):

$${\dot{\theta }}_{i}=\omega_{i}+\lambda \;\sum_{j=1}^{N}\;A_{i,j}\;\sin\;\left(\theta_{j}-\theta_{i}\right),$$ (1)

where *N* is the number of oscillators in the system, *A* is the adjacency matrix of the connectivity and is binary (unweighted), with $A_{i,j}$ denoting either the presence ( $A_{i,j}\neq 0$ ) or absence ( $A_{i,j}$ = 0) of a connection. The diagonal elements of the matrix were equal to 0. The coupling term *λ*, which can take a variety of forms depending on the normalization of the global coupling (Arenas et al., 2008), was set to $\frac{K}{Nd}$ , with *d =* $\frac{1}{N}\sum_{i,j}A_{i,j}$ , the density of the adjacency matrix *A*, in order to mitigate the impact of different densities between networks. K is a constant determining the strength of global coupling between the oscillators.

The presence of noise (Botcharova et al., 2014) or conduction delays (Cabral et al., 2014) is sometimes incorporated into the Kuramoto model to increase its neurobiological realism. Here, for the sake of simplicity, no noise or delays were incorporated.

The degree of synchronisation amongst the oscillators was quantified by the order parameter, *r*, calculated as follows (Equation 2):

$$r\;e^{i\Psi }=\frac{1}{N}\;\sum_{j=1}^{N}e^{{i\theta }_{j}},$$ (2)

where Ψ is the average phase of the oscillators and r describes the global agreement or coherence between the phases of the oscillators. It takes the value of [0,1], with 0 denoting complete incoherence between the oscillators and 1 complete synchrony.

### 2.2 Preserving the metastability profile as a dynamical signature: significance and rationale

Metastability is a concept that has gained much traction in neuroscience and brain dynamical modelling in the last 15 years or so. Whilst there are various definitions of metastability in the literature (Hancock et al., 2023), it is generally agreed that it expresses the concomitant tendencies for dynamical network integration and segregation (Kelso, 2008; Tognoli and Kelso, 2014; Deco et al., 2017; Lord et al., 2017). The significance of metastability in brain dynamical modelling has been underscored by the finding that the optimal working point of various models coincides with the point in the parameter space where metastability is maximised (Cabral et al., 2011; Váša et al., 2015; Deco et al., 2017). Consequently, neuroscientists have hypothesised that the brain at rest optimally functions within a metastable regime (Hellyer et al., 2015; Kringelbach et al., 2015; Alderson et al., 2020). According to the metastable brain hypothesis, metastability provides the necessary state flexibility that the brain requires to timely and “flexibly” respond to external stimuli and to manage the changing coordination needed between different regions to accomplish different tasks (Tognoli and Kelso, 2014).

The most ubiquitous measure of metastability in dynamical brain models has been that of the variability over time of the Kuramoto order parameter (KOP) (Shanahan et al., 2010). Metastability has been shown to be sensitive to structural network alterations, including lesions of single nodes from a model brain network (Váša et al., 2015) and disease-induced changes in mean BOLD signals (e.g., Alderson et al., 2018).

In this investigation, we used metastability as a dynamical signature which, when preserved in a group network, would evidence its representativeness of the cohort. We measured metastability as the STD of the global KOP, which we calculated from Kuramoto simulations of brain networks (see Section 2.6 for the precise model parameters we used). We measured metastability for a wide range of coupling parameters—the entire range needed to drive the network from incoherence to synchrony.

We name the entire curve of metastability values for each of the couplings tested the “metastability profile”. We hypothesise that it can inform us of the network structure and its interaction with a specific dynamic more holistically than simply choosing a specific metric or statistic of the network. This idea is supported by research indicating that network structure may differentially affect the dependence of the synchronisation process on coupling (e.g., Gómez-Gardeñes and Moreno, 2006; Gómez-Gardeñes et al., 2007). For example, hubs in scale-free networks lead to a faster (with respect to K) onset of synchronisation compared to random networks, but they resist global synchronisation for higher K values (Gómez-Gardeñes et al., 2007).

### 2.3 Constructing the candidate networks

To generate a pool of group network candidates from which to determine the one with the least deviation in terms of metastability profile from the cohort, we used uniform consensus thresholding (Heuvel and Sporns, 2011) for the entire range of thresholds from 2.5% (where a link was retained if it was present in at least one subject) to 100% (where a link was retained only if it was present in all subjects in the cohort). The total number of group networks generated was thus equal to the number of subjects in the cohort; in our case, that was 40, the size of our original dataset.

For reference, we also determined the acceptable range of thresholds as per the method proposed by de Reus and van den Heuvel (2013), which aims to make a choice that optimises the trade-off between false positives and false negatives in the group reconstruction (see Introduction). Along with proposing the acceptable threshold range to be 30%–90%, de Reus and van den Heuvel (2013) further suggested that 60% consensus threshold constitutes a suitable choice.

The code implementing uniform consensus thresholding and the method of de Reus and van den Heuvel (2013) was developed in MatLab (Version: 9.8.0.1359463 (R2020a) Update 1).

### 2.4 Identifying the most dynamically representative network

The metastability profile was constructed for each of the group networks from the candidate pool (Section 2.3) as well as the individual subject networks. For each of the 40 group networks constructed by consensus threshold G_T_, with threshold T = 2.5, 5, … 100%, the mean squared error (MSE) was calculated between its metastability profile (*MP*) and that of each of the individuals, *s*_*i*_, as follows (Equation 3):

$$MSE_{T}=\frac{1}{n}\sum_{i=1}^{n}{\left({\text{MP}}_{G_{T}}‐{\text{MP}}_{s_{i}}\right)}^{2},$$ (3)

where n is the number of individual networks considered.

This quantified the average dynamical deviation of each group network from the subjects it purports to represent. The most dynamically representative connectome was then chosen as that which minimised the *MSE*_*T*_—that is, the group network with the metastability profile closest to that of the individual subjects. We called this connectome the “dynamics-based consensus” (DBC). We note that using the MSE versus a higher-order criterion likely strikes a balance between emphasising regions of the curve where the differences are larger without obliterating areas of smaller difference.

### 2.5 Connectome dataset

The structural connectivity matrices from n = 40 healthy adult participants (Kyriazis, 2022) were used (28 females). The participants’ mean age was 44.97+/-3.07 years. The participants were scanned using a 3-Tesla MRI scanner with a 64-channel head coil (Siemens Prisma scanner; Siemens, Erlangen, Germany) with a protocol that included T1-weighted and multi-shell diffusion-weighted sequences. The anatomical data were processed using FreeSurfer 6 (http://surfer.nmr.mgh.harvard.edu/) and parcellated into 84 regions using the Desikan–Killiany atlas (Desikan et al., 2006). The diffusion data were processed using MRtrix3 (Tournier et al., 2019), relying on multi-tissue constrained spherical deconvolution (Jeurissen et al., 2014). Probabilistic tractography was performed using a probabilistic algorithm (iFOD2) and anatomical constraints (Smith et al., 2012), followed by a filtering step (Smith et al., 2013). Finally, connectivity matrices were reconstructed by rigidly aligning the anatomical and diffusion datasets and counting the streamlines interconnecting each pair of regions. The edges were weighted by the number of streamlines connecting two regions. For this investigation, we binarized the networks, with A_*i,j*_ = 1 denoting the presence of an edge between regions *i* and *j* and A_*i,j*_ = 0 denoting its absence. We discarded the information in the weight distribution as only considering the binary connectivity between regions (the existence or absence of an edge), bypassing the issue of the lack of agreement on the best weighting scheme for brain networks (Yuan et al., 2019) and increasing the simplicity and interpretability of the model. Additionally, limiting ourselves to binary connectivity enables us to carry out comparisons with previously published results on the construction of representative group connectomes (de Reus and van den Heuvel (2013); Betzel et al., 2019).

Prior to analysis, the regions of interest (ROIs) covering the cerebellum were removed, resulting in a final structural connectivity matrix of 82 nodes.

### 2.6 Kuramoto simulation parameters

For each realisation of the system, the oscillators were assigned random initial phases in the interval (-π, π) and natural frequencies randomly drawn from a standard normal distribution with a mean frequency of 40 Hz (∼251 rad/s) and a standard deviation of ∼0.16 Hz (1 rad/s). This frequency was chosen because it is often used in published brain network simulations (see Cabral et al. (2011) and Váša et al. (2015), for examples). However, the choice of frequency does not affect the metastability profile since this characterises the relative behaviour of each oscillator with respect to a rotating frame in which the phase average over all oscillators is 0. A small standard deviation was chosen to reduce the likelihood of the results being determined by a particular arrangement of natural frequencies to oscillators. Indeed, it has been shown that microscopic correlations between the structure and dynamics of the network—such as hubs of the network being assigned higher frequencies—can alter the nature of the transition, leading to effects such as an explosive synchronisation in some complex networks (Gómez-Gardeñes et al., 2011). We assumed that a smaller standard deviation would be sufficient to circumvent this possibility, making the results more dependent on the structure of the network. Setting the standard deviation to 0 (i.e., assigning identical frequencies to the oscillators) would not be a viable option as it would abolish the transition, leading to complete synchronisation in the network (Ponce-Alvarez et al., 2015).

Total simulation time per realisation was 100 s, as this was long enough for the order parameter to reach a steady state. The first 50 s were excluded from analysis to discard the transient. The system of equations was solved numerically using Euler integration and an integration time step of dt = 0.001 s; this choice did not affect the results. The range of coupling strengths used was K = 0–3, with a step increment of 0.125 for a total of 25 values. This range of K values was chosen to capture the full transition from incoherence to nearly full synchrony.

Our simulations were implemented using the code made available by Cabral et al. (2014) at https://sites.google.com/site/cvjoanacabral/codes as amended to reflect our choice of *λ* (see Equation 1 and text underneath).

### 2.7 Group connectome construction methods based on structural optimisation

For reference purposes, we also constructed group networks using consistency-based thresholding and distance-dependent thresholding. These were compared to the DBC in terms of how well they represented the individual subjects dynamically (via the metastability profile) and structurally.

#### 2.7.1 Consistency-based thresholding

This method (Roberts et al., 2016) generates a group connectome of a given network density (free parameter that varies between 0 and 1) which retains those links with weights with the lowest coefficient of variation (SD/mean) across the individuals. To implement this method, we used the weighted version of our brain networks as inputs (and binarized the output group network). A single output group network was constructed, with a density set to be equal to that of the DBC for comparison purposes (∼0.62). This density is higher than that used in Roberts et al. (2016) for most measurements (30%). However, such a difference is not unexpected considering that that study dealt with networks with a substantially finer parcellation (513 nodes versus 82 here). The binary density of any network representation of the connectome critically depends on the resolution of the representation; as the resolution becomes finer, the number of regions represented increases, causing an increase in the possible number of network links without an equal increase in the number of actual links. Using an extreme example, in a network where every single neuron is represented by a node, with the edges representing the neuronal processes that make synaptic connections, the density would be extremely low, given that out of 10^22^ possible links that could exist between the ∼8.6 × 10^10^ neurons, only 10^15^ synaptic connections actually do exist (Gerum et al., 2020).

The method was implemented using the code made available by the authors at https://github.com/breakspear/threshold-consist.

#### 2.7.2 Distance-dependent thresholding

To apply this thresholding method (Betzel et al., 2019), the coordinates for the Desikan–Killiany atlas were obtained from the R package brainGraph, version 3.0.0. Using these coordinates, a distance matrix was obtained containing the Euclidean distances between nodes. Although the use of the Euclidean distance may underestimate the true connection length between two brain regions (due to being the straight-line distance), it was chosen for its consistency with the method applied in Betzel et al. (2019).

The algorithm that implements the method divides all edges in the distance matrix (containing the Euclidean distance between the centroids of the connected regions) into linearly spaced bins (here, 41 bins were used) based on their Euclidean distance. The edges in the final group network are then chosen separately for each bin: the mean number of edges *m* across subjects in each bin is calculated, and the *m* most common edges in that bin are retained in the group matrix. This process is performed separately for inter- and intra-hemispheric connections to ensure that the latter are not under-represented; from all three methods used, this step is unique to this particular thresholding technique. The result is a group network with an inter-areal Euclidean distance distribution that closely corresponds to that of the subjects and a density approximately equal to the average subject density. Here, the average subject density is ∼0.59 (STD: ∼0.04), and the density of the distance-dependent network is ∼0.59.

This method was implemented using the code made available by the authors at https://github.com/brain-networks/distance_dependent_consensus.

### 2.8 Network measures

We used a number of graph measures to assess the degree to which structural similarity predicted the dynamical representativeness of the group networks. Specifically, the following local network metrics were calculated: nodal degree, clustering coefficient, betweenness centrality, and eigenvector centrality. Global metrics included network density, modularity (Newman, 2006), average nodal clustering coefficient, and characteristic path length. For the definitions of these metrics, see Betzel et al. (2019). We note that the *average* nodal clustering coefficient, where the nodal clustering coefficient is a measure of the number of triangles relative to triples around a node, has been used before as a measure of the general “cliquishness” of the neighbourhood of a node in the network (Watts and Strogatz, 1998). The choice of some of these metrics was partially informed by literature that associated the features they quantify with metastability, such as the presence of community structure (Friston, 1997; Wildie and Shanahan, 2012) and the eigenvector centrality of nodes (Váša et al., 2015). However, it has been shown that one or a few of these features alone are not enough to generate dynamical behaviour of the magnitude of complexity generated by the human connectome (Zamora-López et al., 2016). As such, we followed an inclusive approach and supplemented the analysis with additional metrics (see Section 3.2.2) that have been widely assumed to be fundamental in terms of describing the organisation of brain networks (Rubinov, 2016; de Reus and van den Heuvel, 2013; Betzel et al., 2019).

All measures were calculated using the Brain Connectivity Toolbox https://sites.google.com/site/bctnet/(Rubinov and Sporns, 2010).

### 2.9 Statistical comparisons of network measures

To compare subject-level with group-level network metrics, we followed the approach proposed by Betzel et al. (2019) and used Kolmogorov–Smirnov (KS) tests to compare the empirical distribution functions of metrics defined at the nodal level as well as the z-score for global metrics.

For each of the above local network metrics and for each of the group networks considered, the KS test statistic was calculated between the group network and each of the individuals. These statistics were then averaged to obtain a mean KS statistic. As the KS test calculates the maximum distance between two cumulative distributions, a lower value was interpreted as closer correspondence between these. Significant differences between mean KS tests were evaluated using either Welch’s *t*-test (when comparing two means) or a one-way ANOVA (when comparing multiple means).

For global network measures, the z-score was employed to compare the difference between each group network and the individual networks, with a small z-score (in absolute value) indicating similarity between group network and individual networks. All statistical tests were implemented using native MatLab functions.

## 3 Results

### 3.1 Validation using ground truth synthetic data

Because the absence of ground truth in empirical networks does not allow for an objective measure of the effectiveness of our method, we constructed a synthetic data set in which the ground truth network was known. The dataset was constructed as follows. Firstly, we randomly picked a subject network from the empirical dataset as a “seed” network (ground truth). Then, after binarizing it, we generated 40 synthetic networks through random additions and deletions of edges. The number of edges modified for each synthetic network was drawn (after rounding) from a normal distribution with a mean and standard deviation derived from the statistics of the Manhattan distances between the individual networks in the empirical dataset. Finally, to ensure that a) all synthetic networks retained some features of the ground truth network, and b) for very high thresholds, the surviving networks would have the density observed in the empirical dataset, we implemented an immutable core by excluding a randomly picked set of 30% of the edges of the ground truth network from the re-wiring process. As will be further discussed in Section 4.7, we wish to stress that, whilst we have tried to capture some quantitative feature of the between-subject variability observed in the empirical dataset, our synthetic data construction process should in no way be construed as a suggestion that in empirical connectomes, between-subject variability arises from some random deviation from a putative blueprint or ground truth network. That is clearly not the case.

We applied our method to the dataset thus constructed, generating 40 group networks by applying consensus thresholding as per Section 2.3. We then identified the DBC by choosing the network that minimised the mean squared error (MSE) between its metastability profile and that of the synthetic networks, as per Section 2.6.

**FIGURE 1:**
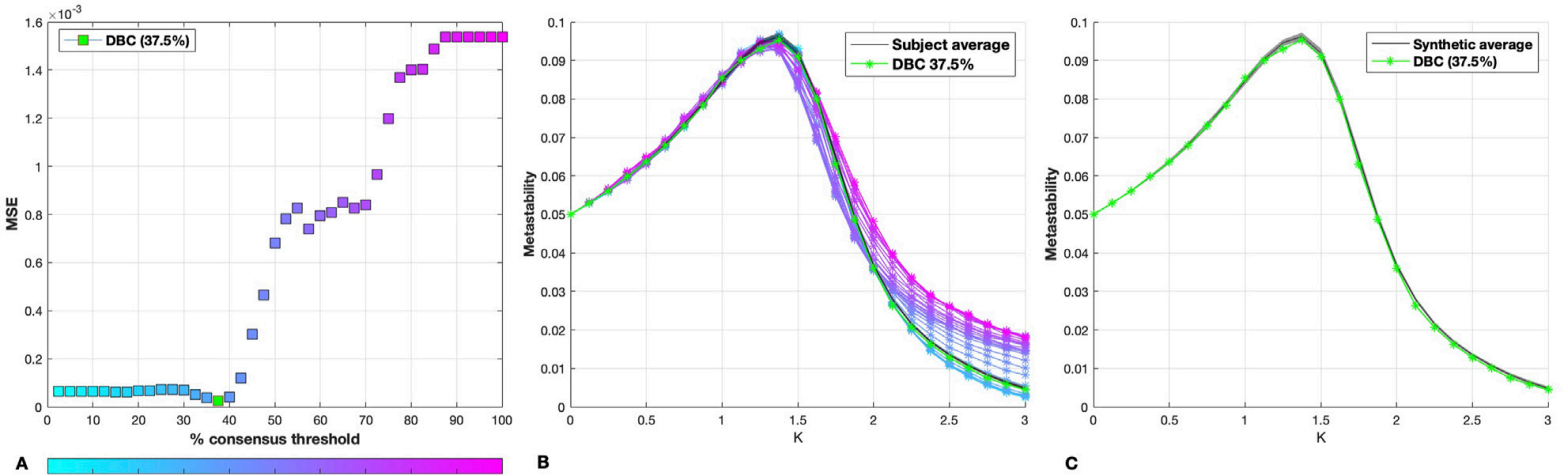
**(A)** MSE between the metastability curve of each percentage consensus group network and that of the individual synthetic networks. The MSE for the DBC connectome is indicated in green. **(B)** Metastability profiles for the DBC (green), consensus-thresholded group networks (blue to pink) and synthetic dataset average (black). The shaded area around the synthetic average corresponds to the standard deviation of the individual synthetic network metastability profiles. **(C)** Synthetic dataset average and standard deviation, with DBC (green).

As shown by Figure 1A, the DBC was found to correspond to the 37.5% consensus thresholded network, with a metastability profile showing excellent agreement with the average of the synthetic dataset (Figure 1C).

**FIGURE 2:**
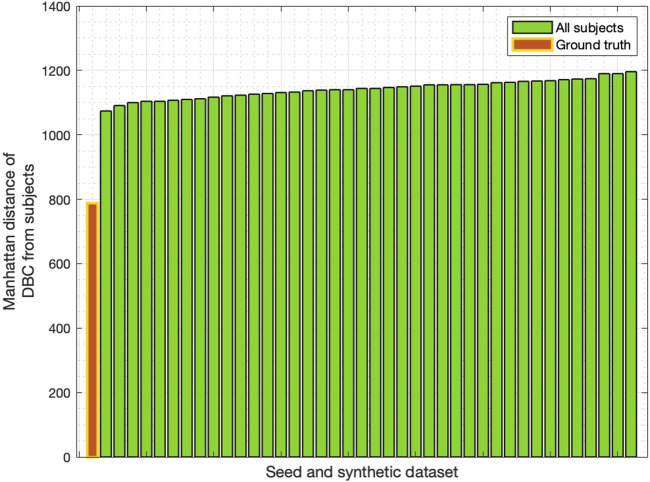
Manhattan distance between DBC and all networks in the synthetic dataset, including the “seed” ground truth network (shown in red). Distances sorted in increasing order.

We then used the Manhattan distance to identify which of the individual networks (ground truth or rewired) was closest to the DBC. As shown by Figure 2, the minimum Manhattan distance was found to be with the ground truth network.

### 3.2 The metastability profile of the dynamics-based consensus (DBC) is a very good fit to those of the individuals

**FIGURE 3:**
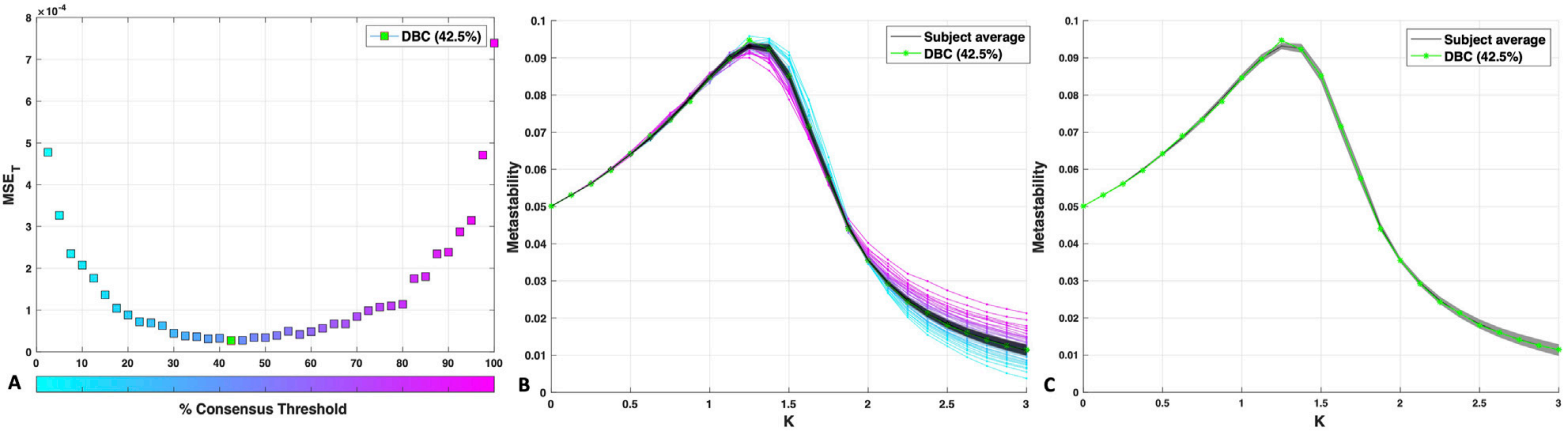
**(A)** MSE between the metastability curve of each percentage consensus group network and that of the individual networks. The MSE for the DBC connectome is indicated in green. **(B)** Metastability profiles for the DBC (green), consensus-thresholded group networks (blue to pink) and subject average (black). The shaded area around the subject average corresponds to the standard deviation of the individual metastability profiles. **(C)** The subject average and standard deviation plotted separately with the DBC (green) to clearly illustrate the quality of the fit.

The DBC network is the group network constructed by consensus thresholding which minimises the mean squared error (MSE) between its metastability profile and that of the individuals (see Methods, Section 2.4). Plotting the 40 group networks constructed in the range 2.5%–100% produced a relatively flat-bottomed U-shaped curve (Figure 3A), with the highest MSEs found for the lowest (2.5%) and highest (100%) consensus thresholds.

A global minimum (the DBC) was identified within the “flatter” region of the MSE curve, corresponding to 42.5% consensus threshold, a group network retaining all edges incident in at least 17/40 subjects (MSE: 0.27 × 10^−4^; Figure 3A). The tight standard deviation around the average of the individual metastability profiles (grey shaded area, Figures 3B, C) indicates high consensus amongst subjects in terms of dynamical behaviour, allowing for an individual average that effectively represents the cohort. The DBC shows a remarkably good fit with the individual subjects’ data average (Figure 3C). The location of the peak at K = 1.25 was maintained in the DBC, although the peak height deviated slightly from the subject average. The peak location is likely to signify an important point for network synchronisation dynamics as it is proximal to the transition from incoherence to synchrony (Wildie and Shanahan, 2012).

It is worth mentioning that the extended flat region covers a range of thresholds (here, 30%–70%, Figure 3A) that is consistent with the acceptable range identified via the method of de Reus and van den Heuvel (2013) (25%–70%). Note too that these results do not qualitatively change upon excluding from the calculation of the MSE a region of the metastability profile with very high similarity to the subjects, from K = 0 to K = 1 (data not shown).

**FIGURE 4:**
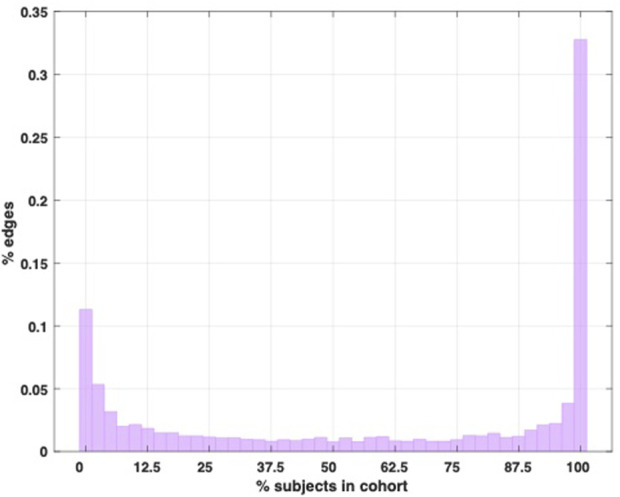
Edge prevalence histogram. This histogram shows the percentage of edges that are shared amongst each percentage of the subjects in the cohort represented in the dataset.

Given the tight standard deviation around the subject average (Figure 3B), we used the edge prevalence amongst the cohort to gain a general overview of structural inter-subject variability by quantifying how many links they have in common. Figure 4 below shows a “prevalence” histogram, illustrating the percentage of links shared by each percentage of the subjects.

Our cohort consists of 40 equally sized matrices *S* encoding the same structure. Therefore, each link *S*_*i,j*_ represents the same connection in each matrix. If *S*_*i,j*_ does not exist in any of the subjects, it is counted in the leftmost bin of the histogram in Figure 4 (corresponding to 0% subjects in the cohort), while if *S*_*i,j*_ exists in *only one* of the subjects, it is counted in the second leftmost bin (corresponding to 2.5% or one subject in the cohort). If, on the other hand, it exists in all 40 of the subjects, it is counted in the rightmost bin in Figure 4. One can imagine overlapping the binary adjacency matrices of the subjects and adding up the 1 and 0 results in each element to produce a matrix with the data plotted in this histogram.

Figure 4 shows that there is a higher degree of global agreement amongst the subjects (links that exist in 100% of the subjects) than absolute disagreement (links that are found in only one subject), and at least 44% of the topology is constant amongst the subjects (since the links in the leftmost bin do not exist in any of the subjects, which also constitutes agreement), suggesting a significant core of conserved edges. Whilst this core may account for the relatively low variability in terms of the subjects’ metastability profiles, it remains true that over half of the network structure is variable amongst the subjects (to different degrees), thus suggesting that the dynamical signature may be somewhat insensitive to some of the structural differences (see also Discussion).

### 3.3 How different is the DBC from the structurally optimised group connectomes?

In this section, we compare the dynamical representativeness of the DBC with that of the other group networks (Section 2.7). We then proceed to assess whether focusing on matching the dynamical behaviour incurred any cost to the structural representativeness of the group network. Finally, we examine whether representativeness in terms of structural metrics predicts the dynamical representativeness of the networks.

#### 3.3.1 Dynamical representativeness of the different group connectomes

We compared the DBC with the group networks constructed using the consistency-based and distance-dependent methods (Section 2.7). An additional comparison was made with the group network obtained by the 62.5% consensus threshold. This threshold was chosen so that it was well within the “safe range” of 25%–70% determined by applying the de Reus and van den Heuvel (2013) model while additionally being close to their more specific recommendation of 60% consensus threshold constituting a suitable choice.

**FIGURE 5:**
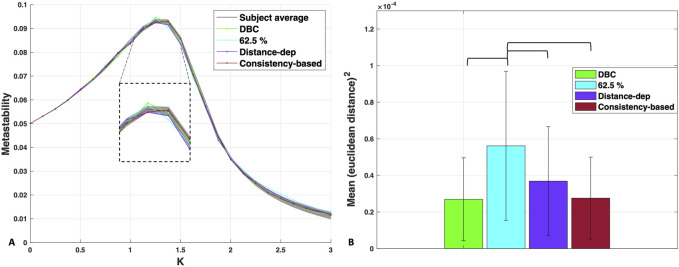
**(A)** Metastability profiles of the dynamics-based consensus, 62.5% consensus threshold group network, distance-dependent and consistency-based group networks, overlapped with the subject average. Grey shaded area denotes subject standard deviation. *Inset:* Focus on the area around the peak. **(B)** Statistical comparison of the mean Euclidean distances squared between the metastability profiles of each of the group networks and those of the subjects. Significance lines for *p* < 0.01, Bonferroni corrected.

Figure 5A shows the metastability profiles of the structurally optimised group networks overlayed on the subject average, with the DBC plotted for comparison. A fit almost as good as that of the DBC is indicated in the plot for those networks, albeit less so for the 62.5% consensus threshold, for which there is an increased mismatch on the right side of the peak. We compared the quality of the fit around the peak region, which is especially important in the process of network synchronisation due to the peak being proximal to the transition (Wildie and Shanahan, 2012). The inset of Figure 5A shows that the two networks constructed via consensus thresholding, the DBC and 62.5% consensus, maintained the same peak location with a slightly increased peak height. In contrast, the peak heights of the distance-dependent and consistency group networks were lower, with the precise values of metastability in these regions being closer to the average subject profile—within the shaded STD region. This may indicate that the structural features maintained by consensus thresholding affect the dynamics differently compared to the distance- and consistency-based thresholds. It is also worth noting that the actual maximum of the consistency-based network is one value after the others, at K = 1.375, which could indicate differences in the pathway to network synchronisation compared to the rest—although at this stage, this remains conjecture (see Discussion for more details).

To obtain a more quantitative estimate of the differences between the group networks in terms of their average distance to the subjects, we statistically compared the mean Euclidean distances squared between the metastability profiles of each of the group networks and those of the subjects (Figure 5B). The results showed no statistical difference between the distance-dependent, consistency, and DBC in terms of the proximity of their profiles to the subjects. The only group network that significantly deviated was the 62.5% consensus threshold, F (3,156) = 8.33, *p* < 0.001, with a medium effect size of *η* ^2^ = 0.1381, indicating that the safe region of representativeness may be narrower than what a visual inspection of the MSE curve in Figure 3A indicated, as well as the safe range of 25%–70% determined by the method in de Reus and van den Heuvel (2013). Overall, these results suggest that selecting for dynamical proximity amongst networks constructed in the absence of distance or weight information achieves an equivalent degree of dynamical representativeness as those networks constructed by primarily utilising this information.

#### 3.3.2 Comparing the structural representativeness of the different group networks

We compared the consistency-based, distance-dependent, and 62.5% uniform consensus network to the DBC in terms of their proximity to the subjects in several local and global network metrics (see Section 2.8).

In terms of nodal metrics, there was no significant difference found between the DBC and the distance- and consistency-based networks. Therefore, choosing a network from consensus thresholding based on minimising dynamical similarity recovered a very similar structure to group networks which were constructed by placing deliberate constraints on the distance distribution or weight variability—at least as far as these nodal metrics were concerned.

**FIGURE 6:**
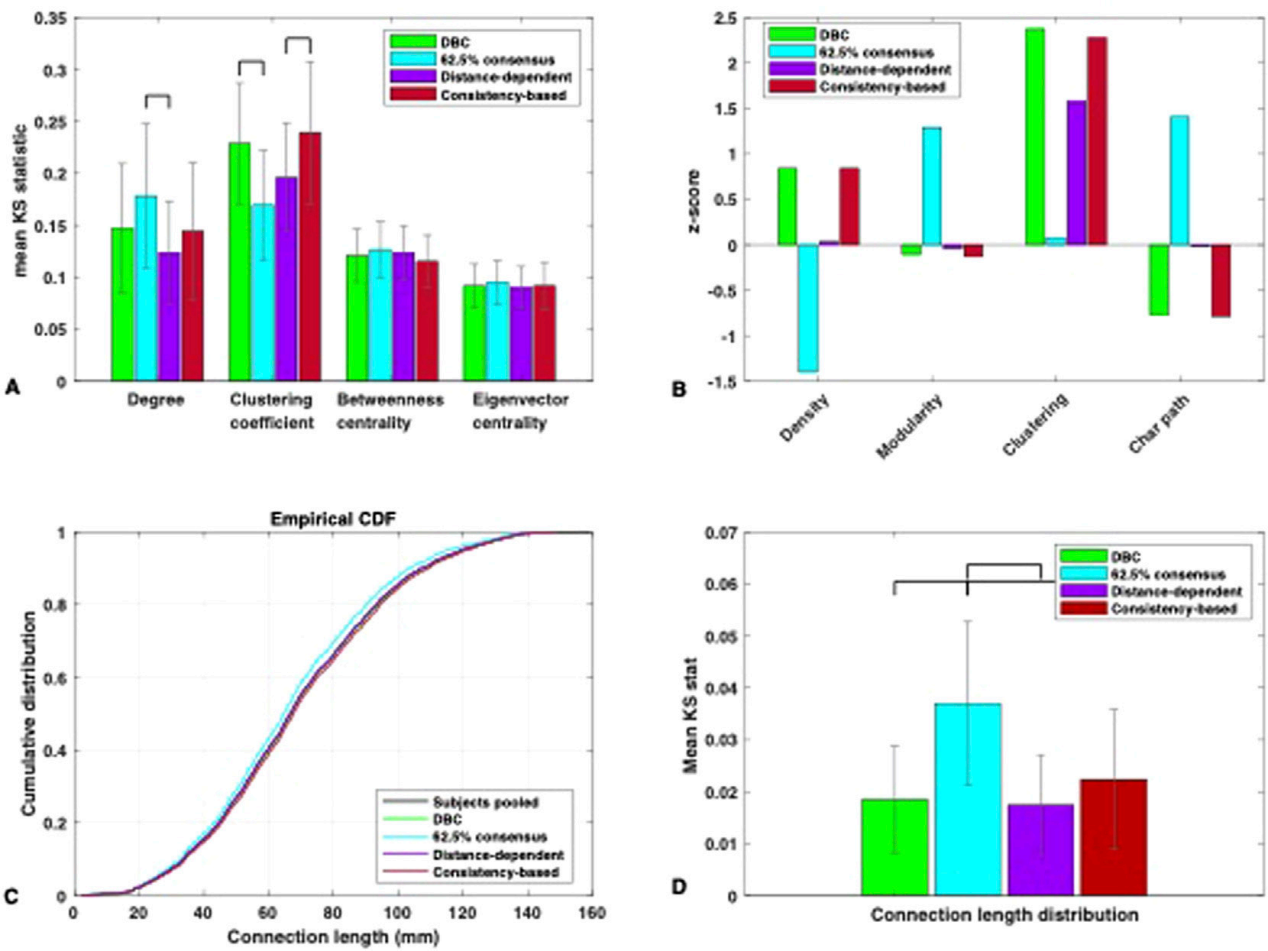
**(A)** Mean KS statistic (and STD) between each group network and the individual networks for the nodal-defined measures of degree, clustering coefficient, betweenness centrality and eigenvector centrality. Significance lines for *p* < 0.01, Bonferroni corrected. **(B)** Z-scores for the global measures of density, modularity, average clustering, and characteristic path length for each group network calculated against the individual networks’ distributions. **(C)** Comparison between the cumulative distributions of inter-areal Euclidean distances of the four group networks and that of the individual networks pooled. **(D)** Mean KS statistic (and STD) between each group network and the individual networks for the inter-areal Euclidean distance distribution. Significance lines for *p* < 0.01, Bonferroni corrected.

With respect to uniform consensus thresholding, there were statistically significant differences associated with the 62.5% threshold—specifically, a medium effect size of *η* ^2^ = 0.092 for the degree, F (3,156) = 5.25, *p* < 0.05, and a large effect size of *η* ^2^ = 0.187 for the clustering coefficient, F (3,156) = 11.94, *p* < 0.001 (Figure 6A). The degree distribution of the 62.5% consensus network significantly deviated from the subjects compared to the distance-dependent network, although not from the others. This was despite there being a statistically significant difference in terms of dynamics, indicating that selecting for dynamical representativeness may place constraints in terms of connectivity that cannot always be captured by differences in the degree distribution. Interestingly, the clustering coefficient distribution was significantly closer to the subjects in the 62.5% consensus network than the DBC. This apparent lack of relationship between dynamics and clustering is consistent with a recent investigation by Stegehuis and Peron (2021) showing that, given a high enough average degree (at least 6)—as is the case for the networks examined here—the dynamics on some networks become insensitive to differences in clustering. Suffice to say that, if the goal is to construct a representative group network by placing *structural* constraints, significant consideration of clustering is not necessary. Finally, there was no significant difference in the betweenness, F (3,156) = 1.38, *p* = 0.25, or eigenvector centrality, F (3,156) = 0.38, *p* = 0.77, between the 62.5% consensus network and the rest, indicating that the dynamical signature chosen in this investigation is more sensitive to potential differences amongst group networks than these two metrics.

The representativeness of the group networks in terms of global metrics is shown in Figure 6B. The distance-dependent network was closer overall to the subjects in terms of global statistics than other networks, having the smallest absolute z-score (<0.1) for nearly all global metrics examined (Figure 6B). The only exception was the average clustering coefficient, where consistently with the nodal clustering distribution, the network with the highest similarity to the subjects was the 62.5% uniform consensus network. This was also the network that deviated the most in terms of all other global metrics, consistently being the furthest to the subjects in terms of dynamics (Figure 5B). These results indicate than when placing constraints on dynamical representativeness, the effects on structure may be more obvious in terms of global metrics than nodal metrics—referring specifically to the 62.5% consensus network. However, there is still a degree of leeway, with optimal dynamical representativeness still allowing for some degree of structural deviation, albeit small.

We used the Euclidean distance matrix (Section 2.7.2) to extract the link distance distribution of each of the group networks (the group networks were used as logical masks on the distance matrix to extract the distances between the nodes with existing links). The representativeness of the networks in terms of inter-areal Euclidean distance distribution is shown in Figures 6C and D. There was no statistical difference between the DBC, distance-based, and consistency-based networks. This interesting result shows that matching dynamical behaviour led to a group network that was just as representative in terms of length distribution as one that explicitly minimised this feature. As far as the consistency-based network goes, Roberts et al. (2016) showed that constructing a group network via this method better represented the length distribution of the cohort versus thresholding via weight (see Introduction), but here we show that it did not demonstrate any advantage in recapturing this feature compared to the DBC.

The only network that differed significantly from the rest in terms of inter-areal Euclidean distance representativeness was the 62.5% uniform consensus network. Fewer long-range links survived in the network, and the retained links were occupied primarily by shorter-range connections (Figure 6C). As discussed in the introduction, this is a direct result of increasing the stringency of the % consensus threshold: in the (real) brain, the probability that a link is present decays monotonically with interareal distance, and this is reflected on reconstructed brain networks across multiple reconstruction methodologies (Alexander-Bloch et al., 2013; Betzel and Basset, 2018). Increasing the percentage consensus threshold increases the minimum enforced agreement for the existence of links, gradually minimizing the permitted variability; if the probability for the existence of long-range links is smaller in the individuals, then given the variability among them, it will be further minimised in a consensus group network. As pointed out in Betzel et al. (2019), this will result in group networks with an overrepresentation of short-range connectivity.

## 4 Discussion

### 4.1 Summary

In this investigation, we chose a group connectome that minimised the dynamical deviation from the subjects from a range of networks constructed by uniform consensus thresholding (the dynamics-based connectome, or DBC). Whether applied to a synthetic dataset with known ground truth or to an empirical dataset, we confirmed that this approach successfully led to the selection of a group network with a dynamical signature that closely fitted that of the subjects in the cohort. In the empirical dataset, selecting another consensus threshold (62.5%) which belonged to the safe range identified by de Reus and van den Heuvel (2013) led to a network that significantly deviated in terms of dynamics, despite the threshold not being substantially removed from that corresponding to the DBC (42.5%). Group connectomes constructed with both distance-dependent consensus (Betzel et al., 2019) and consistency-based consensus (Roberts et al., 2016) —techniques that placed structural constraints regarding the connection length distribution (via the inter-areal Euclidean distance) and the variability amongst edge weights, respectively—did not significantly differ from the DBC in terms of dynamics. In terms of structure, nodal metrics between the distance-dependent and consistency-based group networks and the DBC were not significantly different (Figure 6A). Small differences were seen in global metrics (Figure 6B), with the distance-dependent network being the closest to the subjects—something that did not translate into increased dynamical representativeness.

### 4.2 The implications of preserving dynamical behaviour on the structure of the group network

Our results show that preserving dynamical behaviour with our chosen signature could recover structural information that was not directly accounted for in the methodology. This was reflected in the lack of significant differences in the nodal metric distributions (Figure 6A), which were preserved just as well in the DBC as in both the distance-dependent and consistency-based networks. The inter-areal Euclidean distance distribution (Figures 6C, D) was also preserved equally well in the DBC as in the distance-dependent group network, which specifically optimised for this structural feature. Part of the reason for this could be the low variability amongst the individuals (Figure 4), which is likely a correlate of the high density of these networks. Datasets constructed via deterministic tractography—rather than the probabilistic tractography for the dataset used here—are generally sparser and are unlikely to feature such a high degree of inter-subject consistency.

We note that the density, average clustering, and characteristic path length of the DBC were higher than those of the subjects compared to the distance-dependent network (Figure 6B). The differences in the two latter metrics were most likely an expected effect of the increased density. This relationship to density was further supported by the fact that the consistency-based network, which had a density chosen to be equal to that of the DBC, was different from the subjects in nearly identical ways to the DBC when compared to the distance-dependent network in terms of global metrics. An increased network density implies a higher probability of the existence of short paths (characteristic path length) and closed triangles (clustering). It is noteworthy that our dynamics-based criterion selected for a density that was higher than the average of the subjects: with a chosen threshold of 42.5%, the DBC contained more links than the number existing in at least half of the subjects (on the contrary, the distance-dependent thresholding method preserves the average density of the subjects by construction—Betzel et al. (2019)). Clearly, to replicate a metastability profile that was as close as possible to that of the average of the subjects, some extra links were required, for which there was a slightly lower than average degree of agreement amongst the subjects. The higher characteristic path length (Figure 6B) suggests that these extra links contributed to higher structural network integration. However, the effects of the differences in density were not reflected particularly well on any other metrics, such as modularity (another metric that quantifies the balance between integration and segregation). Overall, the results indicate that preserving dynamics using our metastability signature is an efficient way of recapturing a network with high structural representativeness. Of course, by design, the method also comes with the important implication of dynamical representativeness—a very important characteristic when it comes to brain dynamical modelling studies.

### 4.3 Optimising for structure: principal limitations and lessons from our results

For researchers interested in creating group connectomes by preserving specific aspects of structure, our results suggest that different strategies may yield different combinations of characteristics, and choosing for a specific feature may be risky. This was most obvious in the case of the 62.5% uniform consensus threshold (Figure 6). The principle in de Reus and van den Heuvel (2013), based on balancing the number of false positives and negatives, is parsimonious and sound. However, our results showed that the dynamics of the network significantly deviated (already) by consensus threshold 62.5% and that the differences in nodal and global structure started to become significant.

The central issue with any approach relying on consensus thresholding is the lack of ground truth. In the case of de Reus and van den Heuvel (2013), the acceptable range proposed (30%–90%) may be overly permissive given that we do not have certainty about which of these connections constitute errors or individual variability. We stress the point that the lack of ground truth makes it impossible to conclusively assess whether a particular threshold is too low or too high since we do not know how much actual variation exists in the human connectome. As such, the considerable deviation within this range in terms of both structure (as shown by the authors) and dynamical behaviour (as can be seen in Figure 3A) is problematic. As regards how one should go about choosing a threshold, we note that in the absence of an extra source of information such as the dynamical criterion proposed here, consensus thresholds that deviate from 50% can easily lead to a group network that is no longer structurally representative of the group. For example, the addition or removal of many edges would change density, which in turn would affect many graph metrics (e.g., local and global clustering; path-related measures like characteristic path length and betweenness centrality). A different problem, which nonetheless adds to the issue of selecting an appropriately representative group connectome is that of our incomplete understanding of brain networks in terms of graph analysis, reflected in our current lack of a generative model for structural brain networks. When choosing to optimise for one or more structural metrics, the technique becomes a black box if the knowledge about how the structural features precisely interact is missing—a problem that could be approached using null models (Váša and Mišić, 2022).

Even if the chosen network is a very satisfactory structural match to the cohort, detailed analysis of the structural feature interplay is required if high confidence in the global applicability of the method is desired. Finally, another relevant issue is the large number of pipelines that exist to construct structural connectomes (Maier-Hein et al., 2017), which further increases our uncertainty about which structural criterion should be optimised for in terms of representativeness. What would be the consequences of selecting for specific structural features across different levels of resolution, brain atlases, tractography algorithms, and post-processing steps (see Daducci et al., 2012)?

An example of the interplay between graph properties was seen in our specific networks in terms of the proximity of the group networks to the subjects in clustering coefficient and average clustering (Figures 6A, B). The outlier in dynamical proximity 62.5% consensus threshold network paradoxically showed the highest similarity to the subjects in terms of clustering, a feature for which the dynamically representative networks showed the largest difference from the subjects compared to all other metrics. While this is broadly consistent with Stegehuis and Peron (2021), who showed that clustering did not affect dynamics when the average degree of the network was above 6 (as is the case with all networks used here), considering the issue of structural feature interplay may be more informative. Figure 6B shows the smallest difference in clustering, with the subjects for the 62.5% consensus threshold network accompanied by the highest difference in terms of characteristic path length, suggesting that the degree of “small-worldness” of the network was substantially decreased (Watts and Strogatz, 1998). This would decrease the overall integration in the network and affect its metastability.

Some discrepancies that raise questions about structure–dynamics interactions as well as structure–structure interactions were seen in our results. For example, the betweenness and eigenvector centrality distributions of the 62.5% consensus network did not significantly differ from that of the others, despite significant differences in dynamics, while significant differences in the degree were only seen between the 62.5% consensus network and the distance-dependent network. This is consistent with studies showing that centrality measures can fail to highlight dynamically important nodes in a network (van Elteren et al., 2019; Dablander and Hinne, 2019).

In general, the dynamical network behaviour showed both increased and decreased sensitivity to structural metrics, depending on the metric. The metastability profile demonstrated some tolerance in terms of changes in density, modularity, and characteristic path length, which “broke” for consensus threshold 62.5%. On the other hand, the metastability profile demonstrated significant differences not reflected in the distribution of betweenness, eigenvector centrality, and in some cases, degree (Figure 6A). The question must be asked as to which are the most important structural characteristics for the dynamical behaviour of the networks in this study.

### 4.4 The role of community structure in metastability

Comparison of the shape of their metastability profiles (Figure 5A) shows that metastability for the deviating 62.5% threshold network, which was higher in modularity (Figure 5B), was increased relative to the subjects, and most notably in the region of the metastability profile after the phase transition (on the ordered side). Higher metastability largely reflects higher resistance to full network synchronisation, or “frustration” in network dynamics (Villegas et al., 2014). A toy example of a two-block network model in Villegas et al. (2014) underscores the importance of high inter-modular coherence due to, in part, *structural bottlenecks* in terms of preventing global synchronisation and generating dynamics that are highly metastable. If one hypothesizes perfect synchronisation within the modules/blocks, with differences in frequency only in their interface (in Villegas et al. (2014), that interface consists of only two nodes), the emergence of high metastability can be expected trivially as the two frequency clusters meet one another on the unit circle. Where the phases of the oscillators are represented, the order parameter will be equal to 1, while where they oscillate with a difference of *pi,* the order parameter will be equal to 0. This will give rise to maximum-amplitude fluctuations of the Kuramoto order parameter over time. These fluctuations are what the Shanahan (2010)-derived metastability metric used here and in other studies (Cabral et al., 2011; Cabral et al., 2014; Váša et al., 2015) quantifies; as such, higher modularity translates in a straightforward manner into higher metastability.

As mentioned before, structural modularity can control the balance between segregation and integration in the network, which has been emphasised before in terms of its importance for metastability (Tognoli and Kelso, 2014; Zamora-López et al., 2016; Caprioglio and Berthouze, 2024). However, there are different ways to quantify this balance that were not used in this investigation. Betzel et al. (2019) divided structural connectivity group networks into subnetworks corresponding to the different resting state networks that have been identified through functional connectivity (FC). The finding was that of higher structural connectivity density between resting state networks than within them in the group network produced with the distance-dependent consensus method compared to the uniform consensus method. This characteristic points to a more integrated pattern of connectivity produced by the distance-dependent method—at least with these specific biologically relevant partitions chosen. As discussed in the introduction, the characteristic of real brains to be dominated by short-range connectivity is over-represented when applying consensus thresholding (Betzel et al., 2019). Short-range connections tend to be intra-modular (Bajada et al., 2019). As such, the balance between intra- and inter-modular connectivity is likely to be different in these networks (individual and group networks constructed by uniform consensus thresholding). The difference in the peak height and location of metastability (Figure 5A) may be associated with these differences, which were not reflected on the global metric of modularity (Figure 6B) —with the caveat that the identification of the precise peak location depends on the resolution of the metastability profile, which limits our confidence in how precisely that peak location can be identified. Such differences between the networks could also be biologically relevant.

The biological relevance of our results hinges not just on the methodology of this particular study but also on the universal problem of the lack of ground truth connectome, an issue which affects most, if not all, studies that simulate brain dynamics on top of empirical brain connectivity. As such, regardless of how well a dynamical model performs, it will always be intrinsically limited as a result of the imposed network structure.

### 4.5 Strengths and limitations of our model

Our specific choice of the Kuramoto model has been motivated by several factors, including its simplicity—indeed, it allows for an entire behavioural profile for a network to be traced by tuning a single parameter (the coupling constant, K) —and the success of the model in reproducing empirical data (Cabral et al., 2011; Hellyer et al., 2015; Deco et al., 2017; Nguyen et al., 2020).

It should be clear that the simple implementation we used in this study means that one should not draw biological conclusions from either the metastability profiles or the inferred connectome. The absence of spatial embedding and noise and the discarding of weights remove most of the components used in the studies where the Kuramoto model showed its capacity to replicate real brain dynamics (Cabral et al., 2014; Hellyer et al., 2015; Deco et al., 2017). Regarding the specific use of binary topology, assigning the same weight to all existing connections bypasses elements of the structure that could drastically affect the dynamics. In weighted networks, the edge-weight distribution of a variety of weighting schemes (such as streamlines) tends to be heavy-tailed (Nelson et al., 2023). Such heterogeneity in edge weights, even with a Gaussian (weighted) degree distribution (Ivković et al., 2012), can give rise to complex dynamical phenomena, including “rare-region” effects (Moretti and Muñoz, 2013) and the enhancement of the separation of time scales in the dynamics of different regions (Pang et al., 2021). This could also affect the variability in the dynamics amongst the subjects due to the interactions of the heterogeneous weight distributions with the Kuramoto model. In addition, we considered a relatively tight distribution of natural frequencies. A wider natural frequency distribution would introduce more frustration in the synchronisation process and likely a metastability profile with a wider and possibly later peak (higher K value). This, and also incorporating delays and noise, could potentially amplify the impact of structural differences between the networks on the dynamics. In fact, any of these changes would likely result in a different DBC.

If the inferred representative connectome depends on the chosen dynamic, does it then mean that it has no informative value? On the contrary, we share the view (e.g., Zamora-López and Gilson, 2024) that the interpretability of dynamic brain simulations is contingent on network topology and not necessarily on being abstracted from network dynamics. In other words, in studies involving brain dynamical simulations, any insights will necessarily be conditional on the dynamic being used. In this respect, the value of our approach is that it selects for the network that is most sensitive to the interaction between structure and the chosen dynamics (here, as assessed by the metastability profile being preserved). Our definition of representativeness is therefore to be understood as “representative given the chosen dynamic”.

### 4.6 Strengths and limitations of our dynamical signature

Our rationale for choosing to preserve the entire metastability profile as a dynamical signature was described in Section 2.2 and hinges on the sensitivity of metastability to structural network alterations and, specifically in the case of the Kuramoto model, the fact that network structure differentially affects the dependence of metastability on coupling. Of course, many alternatives are possible, depending on experimenter biases. For example, an alternative choice could be to use a scalar measure, such as the peak of the metastability profile. As mentioned in Section 2.2, one of the factors that has facilitated the emergence of metastability as a dynamical measure in the neuroscience literature has been the coincidence of the optimal working point of various brain dynamical models with the “peak” in metastability (Cabral et al., 2011; Váša et al., 2015; Deco et al., 2017). This finding fits nicely with the critical brain hypothesis (Beggs and Timme, 2012), according to which the brain operates close to a critical point; this characterises the dynamics of systems undergoing a phase transition. Shanahan (2010) observed that metastability peaks in the narrow region *around* the critical transition, thus making it a possible marker of proximity to critical dynamics (although note that it is possible to have metastability away from the critical point, as shown by Caprioglio and Berthouze (2024)). As such, the peak of the metastability profile can be seen as a central indicator of the system’s dynamics, and peak deviations relative to healthy states have been correlated with measures of decreased cognitive capacity (Hellyer et al., 2015; Alderson et al., 2018; Alderson et al., 2020).

Nevertheless, our position remains that the entire metastability profile provides a significantly more comprehensive characterisation of the dynamics emanating from a structural brain graph because the network structure can differentially affect the dependence of metastability on coupling (Section 2.2.). For example, the route to synchronisation of two networks of different structure could be different, with nucleation clusters and oscillator interactions occurring at a different order with regards to coupling (Gómez-Gardeñes 2007). This may mean that a single snapshot of the dynamical state of two networks, even if characterised by the same metastability value, may look very different. However, a dynamical profile that contains a measure of the state of the network for every coupling value has a much higher capacity to discriminate between two different networks than a single number. This can be observed in the results of studies such as Gómez-Gardeñes (2007) and Zamora-López et al. (2016).

Still, the possibility remains that specific parts of the profile may be more informative than the entire curve, and the MSE does not disambiguate between different regions of the curve. Additionally, metastability, as measured here, constitutes a global average of the dynamics of the network and could overlook differences in more localised dynamics. This is a measure of the fluctuations of the global order parameter and it does not provide information regarding the origin of those fluctuations, which could have different functional or—given a more realistic version of the model—biological implications. Finally, the metastability profile could “favour” aspects of network structure that may not have a real impact in a more biologically realistic setting (as per the previous section).

Future research could address the value of different dynamical signatures. In a system of synchronising oscillators, the metrics of local/nodal synchronisation, or even local “metastability” or “vorticity” (Escrichs et al., 2022), give similar information—while also tending to be averaged across the entire network—but could potentially exhibit different levels of sensitivity or be shown to provide complimentary information to metastability in terms of disambiguating between networks. As such, they could be used along with metastability to generate a more comprehensive “profile” (or signature) of network dynamics—ideally upon some type of validation. Other metrics, such as those used in Arenas et al. (2008) to quantify the number of connected components and the size of the largest synchronised component, have been shown to be very informative in terms of tracing the structure-dependent pathway to synchronisation (Gómez-Gardeñes et al., 2007). Such metrics could also be considered to construct a dynamical “signature”.

### 4.7 Strengths and limitations of our validation study

Our validation study showed that the proposed method could recover the ground truth network. This suggests that the metastability profile, which is sensitive to the dynamics of the network, can successfully highlight those characteristics that are critical for identifying the ground truth, even in the presence of variability introduced by a noisy generative process.

It is important to note that, whilst our generative process for creating the synthetic dataset sought to preserve some of the quantitative features of the between-subject variability observed in the empirical dataset, it should be clear that this method does not purport to model the true sources of variability observed in human brain networks. In fact, the notion of the existence of a seed or blueprint network is dubious. Secondly, in addition to intrinsic variability between human brain networks, both acquisition and processing of the signals introduce structural biases that cannot be captured by uncorrelated random perturbations. This difference in noise characteristics is actually reflected in the curve showing the distance between the MSEs of the synthetic and group networks (Figure 1A) which displays a dramatically different shape to that obtained with the empirical dataset (Figure 3A).

### 4.8 On the value of composite measures and a multi-objective optimisation approach to mitigate structural bias

Our results indicate that relying on one or only a few structural metrics could be uninformative and/or misleading. Both the distance-dependent and the consistency-based methods relied on metrics that we have shown can capture a number of different network properties at once. We took this thinking one step further by utilising a measure that, at least theoretically, relies on the capacity of the entire network to produce a dynamical (metastable) behaviour, which should be a result of the integrated structure of the network. It is important to note that our method is not intended as a substitute for the aforementioned structurally based approaches. Rather, by turning to dynamics, we attempt to bypass the issue of selecting amongst different structural criteria which will inevitably carry different biases. This is aligned with the general emergentist idea behind complexity science, that the “whole is more than the sum of its parts”, or indeed very different from it (Anderson, 1972). Specifically in terms of the brain, Park and Friston (2013) observed that the function-to-structure relationship in the brain is degenerate, implying that a (relatively) static substrate can produce a variety of functional patterns. This variety can be missed if one neglects dynamical behaviour. Additionally, given the discussion around the benefits of examining dynamic, rather than static, brain functional connectivity (Hutchison et al., 2013; Elton and Gao, 2015), utilising a measure such as the metastability profile, which reflects fluctuations, can provide more information about the dynamical repertoire that can be generated.

There are other composite measures that, whilst not relying on dynamics, aim to capture the structure of the network as a whole. A measure of this type which incorporates topological features of the network at all scales is the network portrait divergence (NPD) (Bagrow and Bollt, 2019). The NPD is a measure that is based on information theory, built on a graph invariant to allow comparison between networks strictly based on topology. However, using this measure presents some challenges—for example, a comparison between the NPD of two networks can be done fairly only when they have a common average degree (Bagrow and Bollt, 2019). Whilst this is true for some of the group connectomes considered, it is not when considering a wide range of uniform consensus thresholds or indeed between individual connectomes.

A different approach to modelling a representative group connectome could be a weighted graph, where each edge weight would encode the prevalence or probability of the edge occurring in the group. Whilst this would reduce the dimensionality of the representation, its use in dynamical simulations would not allow comparison with those obtained using subject connectomes since the edge weights would represent the probability of occurrence rather than any of the metrics normally used in the construction of human brain graphs, such as fractional anisotropy or number of streamlines (Yuan et al., 2019). A corresponding issue would arise with characterizing such a representation with graph theory-derived metrics. Very frequently present low-weight connections in the individual graphs would have very high weights in the proposed weighted network, underscoring the fact that this network constitutes a different model/representation than that of a graph representing the structure of a single human brain. Whilst such a network would not be a representative network in the sense discussed in our work, it could be more suitable for other types of analyses, such as comparisons of relative edge prevalence between groups.

It may be that a multi-objective optimization approach is a solution in terms of capturing a truly representative network. Given that the brain has evolved to support functional patterns of activity (*i.e.,* dynamical behaviours) and assuming that building generative models of brain dynamical activity is a worthy endeavour, we suggest that the simultaneous inclusion of dynamical measures along with structural characterizations of the network makes for a more powerful approach.

### 4.9 How useful is the concept of a group network (and how possible is its implementation)?

Building a group network is useful in terms of increasing the statistical power and interpretability of studies in neuroscience while decreasing their computational expense. Perhaps the most promising instantiation of this concept is in its extension to building reference brain group networks for developmental or disease states and stages, ideally under an open science framework (Burns et al., 2013). At present, there are important obstacles to this goal. Regarding the nature of the empirical/biological data, there may be more heterogeneity within the “healthy” ”neurotypical” human population than the scientific community appreciates in terms of, for example, the anatomical localization of the neural substrates that support particular functions (Gray and Nachev, 2022). If the heterogeneity is large enough, the notion of a common blueprint becomes more elusive. Additionally, there are many different decisions for the specific research team at each step of the methodological pipeline towards building a human brain network; each of these decisions can have its own consequences on the resulting network. In fact, Kiar et al. (2017) found that even after controlling for reported study variables, there was a large amount of variability amongst structural connectomes from 25 different studies, suggesting that “hidden” variables affect these results. Under such circumstances, it is easy to imagine how a group connectome approach could introduce significant, distorting biases. Perhaps the answer lies in specifying a different approach according to the study or research question. Steps towards the standardization of human connectome pipelines are necessary. The ultimate goal of establishing the human brain connectome like that of the human genome is one of the greatest in science, but, as in genome projects, acknowledging and systematizing connectomic diversity is key.

## 5 Conclusion

Given the capacity demonstrated by a dynamical signature to produce an adequately representative network that could recover aspects of structure for which the technique did not explicitly optimize, the question remains as to what constitutes valid and testable criteria for representativeness. Since the brain is an organ whose role in nature depends on its function, we believe that network dynamics should be part of the solution. Dynamics offers a way to evaluate structure more holistically, as per the spirit of complexity science.

## Data Availability

The data analysed in this study are subject to the following licenses/restrictions. The brain connectomes described and used in the paper are available upon request (MM). The analysis code is also available upon request (EK). Requests to access these datasets should be directed to mancinim@cardiff.ac.uk (connectomes), e.kritikaki@sussex.ac.uk (code).
